# Tinnitus in individuals without hearing loss and its relationship with temporomandibular dysfunction

**DOI:** 10.1590/S1808-86942012000200010

**Published:** 2015-10-20

**Authors:** Aline Albuquerque Morais, Daniela Gil

**Affiliations:** a Speech and Hearing Therapist – Federal University of São Paulo – UNIFESP (Audiology specialist – Medical School of the University of São Paulo -FMUSP); b PhD in Sciences – Federal University of São Paulo – UNIFESP (Adjunct Professor – Department of Speech and Hearing Therapy – UNIFESP). Federal University of São Paulo

**Keywords:** hearing, temporomandibular joint dysfunction syndrome, tinnitus

## Abstract

Research has shown that dysfunction of the temporomandibular joint is often associated with tinnitus.

**Aim:**

to characterize tinnitus in individuals with normal hearing and search for a possible relationship with Temporomandibular Disorders (TMD). Study design: prospective and cross-sectional.

**Materials and Methods:**

the participants included 20 adults of both genders with tinnitus and normal hearing thresholds on audiometry. We studied tinnitus psychoacoustic characteristics and employed the checklist of TMD signs and symptoms from the Tinnitus Handicap Inventory (THI).

**Results:**

the high pitch, continuous and bilateral tinnitus was the most frequent. Upon acuphenometry, the average tinnitus pitch reported by the subjects was 8.6 kHz and the average loudness was 14.1 dBSL. The degree of discomfort caused by tinnitus was mild. We observed that the higher the pitch, the lower was the loudness and the higher was the THI score. We found that 90% of the patients had at least one TMD sign or symptom.

**Conclusions:**

the most common was the high pitch, continuous and bilateral tinnitus; 90% of patients had at least one sign or symptom of TMD and there was no correlation between the tinnitus and acuphenometry, THI and the TMD checklist.

## INTRODUCTION

Tinnitus is defined as an experience in which the individual hears a sound without a corresponding sound stimulus^1^.

This symptom, shared by approximately 25 million Brazilians, affects the auditory pathways and may have numerous causes, such as primary ear disorders, or diseases which secondarily affect the ear – such as metabolic, cardiovascular and neurological; pharmacological, psychiatric and dental disorders^2^.

Tinnitus is a very frequent symptom in individuals with temporomandibular dysfunction (TMD)^3^, ^4^.

The most frequent otological symptoms in patients with TMD are: ear fullness, tinnitus and otalgia, and the first prevails over the others^5^.

Although numerous studies associate tinnitus with TMD, a cause and effect relationship between them is yet to be proven. Nonetheless, it is a fact that such symptom is more frequent among individuals with TMD than those in the general population^3^, ^4^, ^5^, ^6^, ^7^, ^8^, ^9^.

Notwithstanding, in some studies which showed a greater prevalence of TMD signs and symptoms in tinnitus patients, they did not do any audiological evaluation or did not exclude those individuals with hearing loss from the sample and, therefore, did not rule out the otological etiology of the tinnitus^10^, ^11^.

Thus, the present study aimed at characterizing tinnitus in normal-hearing individuals and search for a possible relationship with Temporomandibular Dysfunction (TMD). Our specific goals were:

a) To correlate the Temporomandibular Dysfunction checklist with the results from the *Tinnitus Handicap Inventory* (THI) and the tinnitus psychoacoustic characteristics.
b) To characterize the tinnitus frequency and intensity by means of the individuals self-reporting and acuphenometry.
c) To characterize the impact tinnitus has on the quality of life of the individuals by means of the THI.

## MATERIALS AND METHODS

This is a cross-sectional contemporary cohort study, analyzed and approved by the Ethics in Research Committee of our Institution, under protocol # 1440/09.

All the individuals who participated in the study signed the Informed Consent Form.

The patients were recruited at the Tinnitus Ward of our Institution.

Eligibility criteria included:

• Age between 18 and 55 years;
• Both genders;
• Hearing thresholds lower than or equal to 25 dBHL, between 250 and 8,000 Hz;
• No past of otitis media and/or ear surgery;
• No evidence of cognitive and/or neurological involvement.

Based on these criteria, we selected 26 individuals. However, only 20 could be included in the series. Of the six individuals taken off the study, two did not come for data collection, three did not complete all the procedures and one reported he was being treated for otitis.

All the individuals were submitted to the following procedures: inspection of the external acoustic meatus; interview to investigate the person's general health status – aiming at identifying tinnitus-associated disorders such as diabetes; audiological and otological past -exposure to noise, middle ear infections, dizziness, ear surgeries; check for laterality; tinnitus type and duration; identification of the tinnitus pitch and loudness by means of acuphenometry and deployment of a checklist of TMD signs and symptoms, which was created by the researcher, based on data from the literature, talks with professionals in this field (dentists, speech and hearing therapists specialized in orofacial movement), in order to search for a possible relationship between tinnitus and TMD^12^, ^13^, ^14^, ^15^.

The *checklist* involved the following issues:

1. Asymmetrical opening movement?
2. Click sound?
3. Cracking sound?
4. Reduction in mandible movement amplitude?
5. Fatigue in mastication muscles?
6. Pain in mastication muscles?
7. Parafunctional habits (bruxism or tightening)?

The idea behind having a checklist is to enable the clinician to identify patients with a higher likelihood of having TMD and, thus, better and more correctly refer patients. Of the seven questions which make up the checklist, two (questions 1 and 4) were answered exclusively based on the assessment carried out by the researcher and five were based on the report from the individual (questions 2, 3, 4, 5 and 7).

We considered it as asymmetrical mouth opening movement (question 1), when we noticed a lateral shift followed by a return to the midline – a “C” movement. The mouth opening limitation (question 4) was checked using a digital pachymeter, and considered as a present symptom when smaller than 40mm^16^. As to the joint noises (questions 2 and 3), the crackling was differentiated from the clattering sound as a rough noise, like bone scratching over bone. The other questions, 5, 6 and 7, were presented to the patients in the above-mentioned format.

The data was plotted in an Excel spreadsheet for later statistical analysis, which counted on the following statistical tests:The Mann-Whitney test was used to check the relationship between the type of tinnitus (low or high) and the *Pitch*, *Loudness*, THI and *Checklist* parameters; the chi-squared test was utilized in order to investigate the difference in the distribution of TMD signs and symptoms in the *Checklist*; and the Pearson's correlation (c) was used to investigate the correlation between *Pitch* × *Loudness* X THI X *Checklist* – in such a way that | c | < 0.40 means a weak correlation; if 0.40 < | c | < 0.70 it means a moderate correlation; if 0.70 < | c | < 0.90 this means a good correlation and if | c | > 0.90 means optimum correlation.

The level of significance (*p*-value) established for this study was 5% or 0.05. The results with statistically significant difference are stressed with the asterisk (^*^).

## RESULTS

The descriptive analysis of the interview results showed that 70% of the individuals in the study were females; while 30% were males. The mean age of these individuals varied between 20 and 55 years, with a mean of 32.1 years, characterizing a sample of young adults.

In order to help on the analysis of the results, the examiner classified the types of tinnitus reported by the individuals in two categories: high and low, corresponding to 75% and 25% of the sample, respectively. As to tinnitus duration, 90% of the individuals had continuous tinnitus; and 10% reported having intermittent tinnitus. Most of the individuals (60%) had bilateral tinnitus, 20% in the right ear, 5% in the left ear, and 15% perceived the tinnitus in their heads.

Based on Table 1, we notice that the individuals classified their tinnitus as high and with a mean *loudness* of 14.1 dB above their hearing thresholds.

**Table 1:** Descriptive values for *pitch* (in kHz) and *loudness* (in dBSL) obtained from the acuphenometry.

	PITCH	LOUDNESS
Mean	8.6	14.1
Median	9.0	10.0
Minimum	0.25	5.0
Maximum	16.0	50.0
St. Deviation	5.5	10.6

n	35	35

n = number of individuals. St. Deviation = standard deviation.

We observed that the THI mean total score was 25 points (Table 2); which led to classifying the tinnitus as mildly bothersome to most of the individuals in the present study.

**Table 2:** *Tinnitus Handicap Inventory* (THI) descriptive values.

	THI (total score)
Mean	25.0
Median	17.5
Minimum	0.0
Maximum	74.0
St. Deviation	21.3

n	20

n = number of individuals. St. Deviation = standard deviation.

Table 3 showed that the mean TMD signs and symptoms in the study individuals were 2.2, which said that the individuals, in average, had two affirmative answers in the *checklist.*

**Table 3:** TMD signs and symptoms *checklist* descriptive values.

	*CHECKLIST*	
		(total score)
Mean		2,2
Median		2,0
Minimum		0,0
Maximum		5,0
St. deviation		1,6

n		20

n = number of individuals. St. Deviation = standard deviation.

We found a statistically significant difference for Question 1, characterizing the asymmetrical opening movement as the most frequent sign of temporomandibular joint dysfunction in this group of individuals (Table 4).

**Table 4:** TMD signs and symptoms distribution.

	Question 1		Question 2		Question 3		Question 4		Question 5		Question 6		Question 7	
	n	%	n	%	n	%	n	%	n	%	n	%	n	%
No	6	30.0	11	55.0	15	75.0	19	95.0	15	75.0	16	80.0	14	70.0
Yes	14	70.0(*)	9	45.0	5	25.0	1	5.0	5	25.0	4	20.0	6	30.0

Total	20	100.0	20	100.0	20	100.0	20	100.0	20	100.0	20	100.0	20	100.0

n = number of individuals.

* *p*-value= 0.05.

Based on Table 5, we notice that there was a statistically significant correspondence between the *pitch* established in the acuphenometry and that reported by the individual.

**Table 5:** Type of tinnitus, by the comparative analysis between high and low, concerning the following parameters: *pitch*, *loudness*, THI and *checklist*.

		TYPE		Mann-Whitney test (*p*)	Results
		HIGH	LOW		
PITCH	Mean	10,47	3,03	<0,001^*^	High > Low
	Median	10,00	0,25		
	St.Deviation	4,61	3,74		
	N	26	9		

LOUDNESS	Mean	11,35	21,11	86	High = Low
	Median	10,00	20,00		
	St.Deviation	7,008	15,366		
	N	26	9		

THI	Mean	26,87	19,20	396	High = Low
	Median	17,00	18,00		
	St.Deviation	21,92	17,92		
	N	30	10		

CHECKLIST	Mean	2.07	2.60	177	High = Low
	Median	2.00	3.00		
	St.Deviation	1.68	1.08		
	N	30	10		

*p-value= 0.05.

n = number of individuals.

St. Deviation = standard deviation.

As far as *loudness* is concerned, there was a trend towards greater responses for low sounds, in other words, the lower the tinnitus, the greater its *loudness*.

There was a significant correlation between *pitch* and *loudness* and *pitch* and THI, that is, the higher the *pitch*, the lower the loudness; and the higher the pitch, the greater the individual's score in the THI (Table 6).

**Table 6:** Correlation between the parameters: *pitch*, *loudness*, THI and *checklist*

		PITCH	LOUDNESS	THI	CHECKLIST
PITCH	Pearson's correlation	1	−.505(**)	.501(**)	23
	Sig. *(p)*	.	2	2	897
	n	35	35	35	35

LOUDNESS	Pearson's correlation	−.505(**)	1	−174 318	75 0.67
	Sig. *(p)*	2	.	318	0.67
	n	35	35	35	35

THI	Pearson's correlation	.501(**)	−174	1	−62
	Sig. *(p)*	2	318	.	702
	n	35	35	40	40

CHECKLIST	Pearson's correlation	23	75	−62	1
	Sig. *(p)*	897	0.67	702	.
	n	35	35	40	40

n = number of individuals.

** Significant correlation at 0.01.

The remaining correlations were not significant. The Checklist parameter was not correlated to any of the other parameters.

Table 7 showed that the correlation between the parameters analyzed was weak (c) < 40 and not significant, showing that the THI was not sensitive vis-à-vis the complaints associated with TMD.

**Table 7:** Correlation between the THI and the *checklist*.

	CHECKLIST	
	Pearson's correlation	−62
THI	Sig. (*p*)	702
	n	40

n = number of individuals.

## DISCUSSION

Concerning the sample's profile, we could notice that among the 20 individuals, 14 (70%) were females and six (30%) were males. A greater incidence of women with tinnitus and normal hearing was reported by different authors^16^, ^17^. In other studies, the researchers did not find differences between the genders^18^, ^19^. The fact that women more frequently look for health care may have contributed to the results found^20^.

As far as age is concerned, we noticed that the mean age of the individuals in this study was 31.2 years, characterizing them as young adults. This finding disagrees with most of the studies involving individuals with tinnitus, since they usually include older individuals^17^, ^21^. This divergence probably happened because the studies cited involved individuals with hearing loss, often times, stemming from presbycusis.

We must stress that the present study had 20 individuals matching the proposed eligibility criteria, considering the individuals who were taken off because they did not finish the evaluations. Thus, results must be interpreted carefully, especially concerning their generalization.

As to the tinnitus characteristics, most individuals reported having a high pitch tinnitus, continuous and bilateral; concerning the acuphenometry, we noticed that the individuals classified their tinnitus as high and with a mean loudness value of 14.1 dB above their hearing thresholds (Table 1). These findings corroborate those from numerous studies in the literature^16^, ^21^, ^22^. Tinnitus laterality was the only tinnitus-related aspect which had differences vis-à-vis the literature, since in the present study we found bilateral tinnitus in 60% of the individuals, unilateral in 25% and head-located tinnitus in 10%; knowing that other authors^22^, ^23^ reported more unilateral tinnitus in normal-hearing individuals.

The mean THI score of the individuals was 25 points, classifying the tinnitus as mildly bothersome (Table 2). This finding was in agreement with the ones found in the literature^24^, ^25^.

We noticed that the mean score of the individuals in the TMD Checklist was 2.2 points (Table 3). Such finding was in agreement with the literature, since there were different TMD signs and symptoms in tinnitus individuals^12^. The authors of the study cited found that almost one quarter of the patients reported feeling fatigue in their mastication muscles and one third of them reported having clattering. We did not find other studies which investigated TMD signs and symptoms in tinnitus individuals. Most of the individuals in the present study (90%) had one or more positive points in the *checklist* and 60% had two or more points. Only two individuals did not have TMD signs and symptoms. TMD-related symptoms encompass 40% to 60% of the adult population in the United States, being more prevalent among young adult females^26^. Thus, in order to check whether there is a greater frequency of TMD signs and symptoms in tinnitus patients, it would be necessary to compare it with a control group of individuals without tinnitus.

As far as TMD signs and symptoms are concerned, we noticed that the asymmetrical opening movement was the one most frequently found in the present study, being statistically significant in relation to the others (Table 4). Joint noises, such as crackling and clattering, were present in 45% and 25% of the individuals, respectively; and 30% of the individuals had some type of parafunctional habit, such as bruxism or tightening. These findings are not in agreement with the literature, since pain in the region of the mastication muscles was the most frequent TMD sign reported by individuals with tinnitus^12^. Such divergence may be associated to the fact that the questionnaire employed by the authors of the cited study did not involve an investigation concerning mouth opening asymmetry. Notwithstanding, in the same studies, the authors reported the presence of joint noises as the second most frequent sign.

Analyzing Table 5, we notice that the individuals knew how to coherently express the frequency they felt their tinnitus had. We did not find studies comparing the type of tinnitus reported by the individual and the pitch established upon acuphenometry.

Moreover, there was a significant trend concerning loudness and the type of tinnitus, in such a way that, the lower the tinnitus, the greater the loudness (Table 5). We did not find studies associating the type of tinnitus with the feeling of intensity.

In the present study, we also noticed that, the higher the pitch, the lower the loudness; and, the higher the pitch, the greater the individual's THI score (Table 6). These findings are in disagreement with the literature, since we did not find a relationship between the tinnitus *pitch* and THI performance^23^.

There were no statistically significant correlations between the TMD checklist and the other parameters analyzed on Table 6. Thus, it was not possible to identify a type of tinnitus suggestive of, or characteristic of TMD. Some authors stated that the tinnitus characteristics could indicate its etiology. One study carried out with TMD individuals reported the presence of otological symptoms in the patients and found that these individuals' tinnitus was usually moderate, of high frequency and sporadic^10^, which is in partial agreement with the findings of the present study, especially concerning the tinnitus frequency.

Although the individuals had TMD signs and symptoms, the THI score was not sufficiently high to be statistically correlated with the checklist created (Table 7).

## CONCLUSIONS

Based on the critical analysis of these results, one may conclude that the tinnitus individuals with normal hearing in the conventional frequency range assessed in the present study:

• Had tinnitus of high pitch, continuous and bilateral;
• Had a mild impact in their quality of life;
• Had one or more signs of temporomandibular joint dysfunction; and asymmetrical mouth opening movement was the most frequent;
• There was no relationship between tinnitus and acuphenometry, THI and the TMD checklist.
